# Tooth-Inspired Tactile Sensor for Detection of Multidirectional Force

**DOI:** 10.3390/mi10010018

**Published:** 2018-12-29

**Authors:** Nurul Adni Ahmad Ridzuan, Norihisa Miki

**Affiliations:** Department of Mechanical Engineering, Faculty of Science and Technology, Keio University, 3-14-1 Hiyoshi, Kohoku, Yokohama, Kanagawa 223-8522, Japan; miki@mech.keio.ac.jp

**Keywords:** tactile sensor, bio-inspired, tooth, biomimetic, force sensor, three-dimensional

## Abstract

The anatomy of a tooth was the inspiration for this tactile sensor study. The sensor consisted of a pole that was fixed in the middle of an acrylic base using a viscoelastic silicone elastomer. Four strain gauges were fixed three-dimensionally around the pole to detect its movement, which was formed in a single step in the assembly. When the load was applied to the side of the pole, the strain gauges were bent or released, depending on the direction of the applied load and the position of the strain gauges. The sensor device had the sensitivity of 0.016 mm^−1^ and 0.313 N^−1^ against the resistance change ratio. For the load detection experiment, a consistent pattern of full sine-curve, with a constant resistance change for the angles, was obtained for all of the four strain gauges, which confirmed the reliability of the sensor device to detect the direction of applied load. The amplitudes of the resistance change ratio remained to be consistent after loading-unloading processes at the frequency of 0.05–0.25 Hz.

## 1. Introduction

Previously, many tactile sensors were designed to function like skin, as reviewed in the literature [1,2,3,4,5]. Researchers and developers took the anatomy of glabrous skin or hairy skin as the inspiration for their sensor designs. Motivated by the remarkably unique surface of the glabrous skin type, they built ridges on the tactile sensor’s surface to increase its sensitivity for shear and normal force detection [6,7], curvature discrimination [8], and roughness and hardness detection [9]. They also found that the skin was more functional with the help of hairs on its surface [10]. Thus, they applied the use of hairy skin to their tactile sensor’s surface design and were able to detect a wide range of pressures because the “hair” was sensitive to small pressures while the “skin” was used to detect large forces [11]. Whiskers, a type of hair for animals such as mice and cats, were also imitated since several reports had reported use of vibrissae-like sensors for gas flow detection [12] and surface texture discrimination [13,14,15]. In addition to the exterior, the skin’s interior structure was also used as inspiration, particularly the tactile receptors, which were imitated to augment the skin-type tactile sensor [16]. Many tactile sensors mimicked the papilla directly beneath the skin’s epidermis, in order to detect changes in an object’s surface shape [17,18]; the distribution of sensory receptors in detecting pressure, temperature [19], vibration, and sliding [20]; and the nerves connecting the skin to the central nervous system in the signal processing systems [21]. 

One of the potential applications of this type of tactile sensors is in minimally invasive surgery (MIS), which requires the sensors to detect the hardness or softness of the organ tissues, as well as the multidirectional force, sometimes in a narrow space and cavity. A forefinger-like sensor, with a cone-shaped tip at the edge of a cantilever, was proposed to detect the force and elasticity [22], along with a palpation probe to detect the deformation of the probe’s tip [23]. To scan the surface of interest, a ball-point pen-like capacitive tactile sensor containing three-dimensional liquid metal electrodes was developed [24,25].

**Figure 1:**
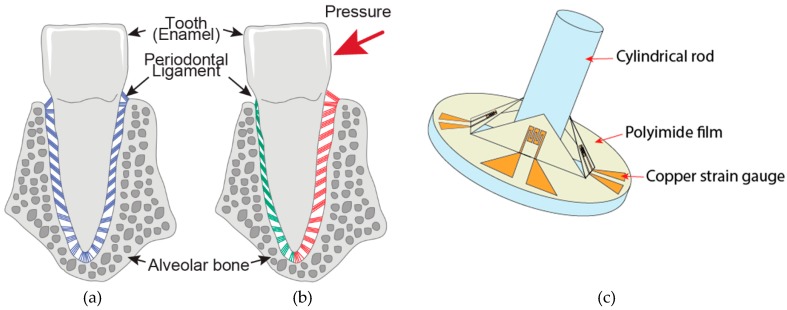
Movement of the periodontal ligaments beneath a tooth (**a**) in resting state and (**b**) with pressure applied. The green and red ligaments indicate compressed and stressed states, respectively. (**c**) Schematic image of the tooth-inspired tactile sensor.

This paper proposes and demonstrates the design and fabrication of a tactile sensor inspired by a tooth’s innate ability to detect the direction and strength of pressure applied on its enamel surface. Periodontal receptors, which located around the root of the tooth, detect tooth movement through the deformation of periodontal ligaments, which are a kind of fibrous tissues that connect the tooth and the alveolar bone. The slight dislocation of a tooth in its socket compresses the periodontal ligaments on the pressure side of the tooth, or stretches the periodontal ligaments on the tension side [26]. The cylindrical shape of the tactile sensor enables the sensor to work in a narrow space. The anatomy of a tooth and the schematic image of the proposed sensor is illustrated in Figure 1.

It is apparent that a tooth’s structure has often been the inspiration for tactile sensor designs, such as the many force sensors designed with a pole, post, probe or mesa structure. Similar to the whisker type of sensor device, the tooth-inspired tactile sensor does not apply forces directly to the sensing elements, unlike the ones inspired by skin. Rather, in this type of sensor, force applied to the sensor’s moveable part allows the movement of said part to trigger the sensing elements, like the strain gauges, attached to it. For example, a whisker sensor for underwater induced vortex detection [27], which has quite a similar design with our proposed sensor, was built using three-dimensional printing technology, with a diameter of 8 mm and length of 160 mm. For MIS application, a small-scale sensor inspired by a hair cell was made, with a diameter of only 420 μm [28], a detectable shear force range below 5 mN, and a post’s maximum displacement of only 15 μm. Building a mesa structure [29] or a probe [30] on a flat sensor element was proven to increase the detectable shear force range. All of these sensors are similar to our proposed sensor in the use of a protruding structure, such as pole, post, probe, or mesa structure. However, unlike the usual whisker type sensors with flat sensing elements, the proposed sensor included a three-dimensional sensing element structure, just like the periodontal ligaments of the tooth. Furthermore, unlike some whisker-like sensors where the protruded structure was glued onto the flat surface, the proposed sensor was inserted and glued inside a hole; this was to set a limit in the displacement of the pole and avoid over-bend, which can cause a destructive effect on the durability of the sensor device itself.

## 2. Design 

**Figure 2:**
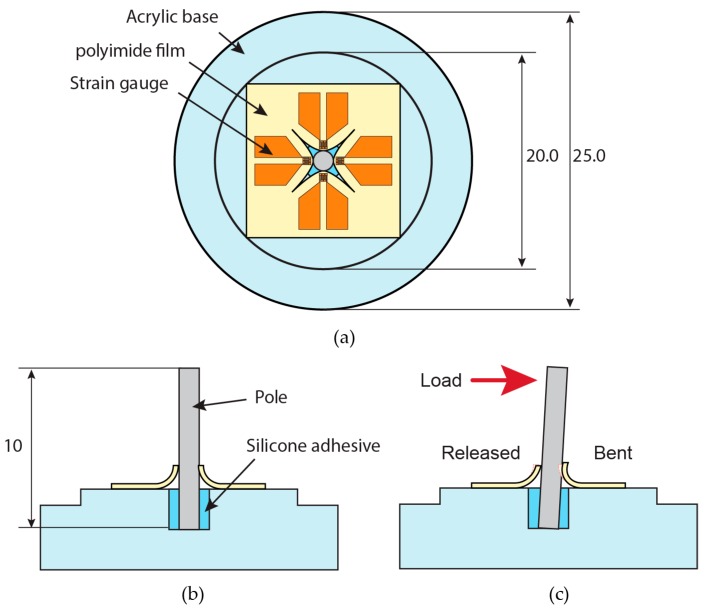
(**a**) Design of the proposed device, top view (above) and (**b**) cross-sectional side view (below). All dimensions are in mm. (**c**) When the load is applied to the pole, one side of the strain gauge is released and the one on the other side is bent.

With a tooth as inspiration, in this research, the sensor was designed to have four strain gauges bent around a stainless-steel pole, as depicted in Figure 2. The pole, which acted like a “tooth”, was attached to an acrylic base, or the “alveolar bone” for the sensor device, using a flexible silicone adhesive material, so that it could be moved back and forth and side to side, for a full 360° range of motion. When the pole was moved in one direction, the strain gauges around it representing the periodontal ligaments in the tooth anatomy were bent more, or released from the initial bending, depending on the direction of the movement. The bending of the strain gauge made the length shorter, thus the resistance decreased. Strain gauge released from the initial bending became longer, so the resistance of the strain gauge increased. The resistance of the strain gauges attached to the sensor device was measured simultaneously during the load application. Different direction of load caused a different pattern of bending or releasing of each strain gauge, thus resulting in a different pattern of resistance change in the strain gauges.

Even the sensor design was simpler and less complicated compared to developing small mechanical springs to attach the pole to the base of the sensor device, using flexible silicone adhesive material as the glue for the center pole; this would surely cause hysteresis in the sensor’s output due to the material’s viscoelastic properties. Therefore, simultaneously measuring the resistance changes of all four strain gauges to deduce the applied force and its orientation compensated for the hysteresis, thus making the sensor output measurable and more reliable.

So far, the proposed sensor device was designed to detect shear force from the sides of the center pole. The base of the center pole touched the acrylic base, i.e., there was no space between the pole and the base. Thus, normal pressing or pulling force from the tip of the pole did not trigger the movement of the strain gauge. 

## 3. Fabrication Process 

**Figure 3:**
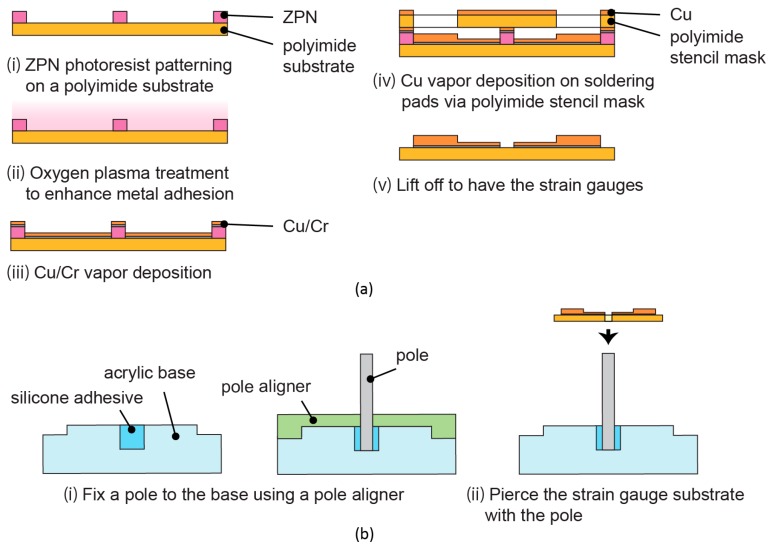
Fabrication process of the device. (**a**) Lift-off process for the making of the strain gauges on polyimide film and (**b**) the assembly process of the base, pole, and strain gauge.

One of the notable characteristics of the sensor is its three-dimensional structure, i.e., a standing pole and bent flexible substrates on which strain gauges reside. The assembly of the structure was a challenge. We came up with the idea of fixing the pole first and then piercing through the center of the flexible substrate with the pole. The position of the pole was elastically balanced, and the contact was assured. The fabrication process is summarized in Figure 3.

The device consisted of an acrylic base, a stainless-steel pole, and four strain gauges. A circular acrylic plate, 5 mm thick and 25 mm in diameter was machined, including a hole, 2 mm in diameter and 2 mm in depth, at its center. The outer corner of the acrylic plate was machined to be the jig of a pole aligner. The pole aligner was also made from an acrylic plate. 

**Figure 4:**
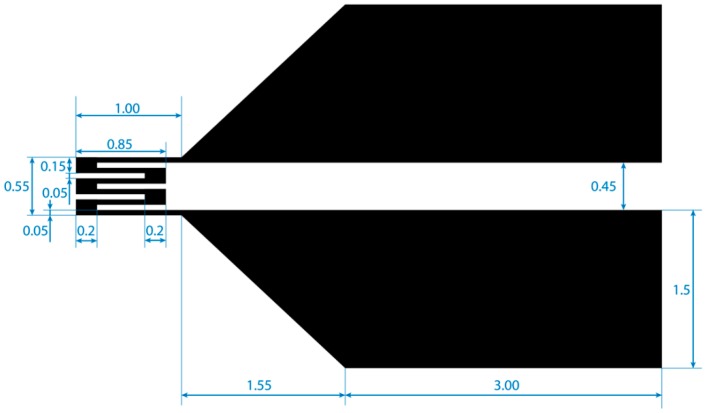
Dimension of a strain gauge in mm.

The strain gauges were made by a lift-off process. The dimension of the strain gauges is shown in Figure 4. First, a polyimide film with the thickness of 50 μm was set on a glass substrate with Kapton tape and the surface was cleaned with isopropyl alcohol. An appropriate amount of 100% wt hexamethyldisilazane primer (OAP, Tokyo Ohka Kogyo Co., Ltd., Tokyo, Japan) was applied to the polyimide film’s surface to ensure proper adhesion between the polyimide film and the photoresist. Next, 1 mL of ZPN 1150-90 photoresist (Zeon Corp., Tokyo, Japan) was spin-coated onto the film. The film was prebaked for 90 s on a 100 °C hotplate and then left at the room temperature to be cooled down. A photomask for negative type photoresist was set on the photoresist film before the photoresist was exposed to ultraviolet (UV) light. After that, post-exposure bake was conducted for 60 s on a 100 °C hotplate. The film was left in a room temperature for 10 min before the development process of the photoresist was done using tetramethylammonium hydroxide solution (NMD-3 2.38%, Tokyo Ohka Kogyo Co., Ltd., Tokyo, Japan). 

**Figure 5:**
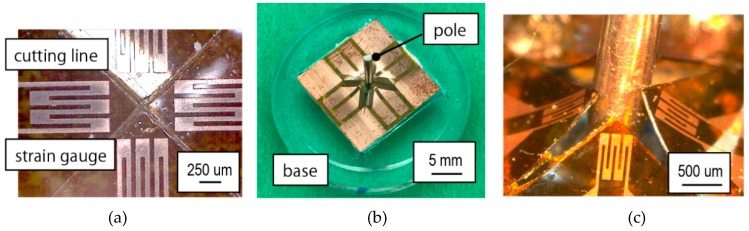
Photographs of (**a**) the fabricated strain gauge, (**b**) the assembled device, and (**c**) deformation of the polyimide substrate with strain gauges when load is applied.

Metals adhere poorly to polyimide film’s surface, and gas plasma treatment has been proven to be the solution for this problem [31,32]. After the development of the photoresist, the film was treated with oxygen plasma to roughen the surface and thus promote the adhesion of metal film to the polyimide film. Chromium (Cr) was then vapor-deposited on the oxygen plasma-treated surface at 35 nm thickness. Here, Cr acted as the contact metal. After that, 110 nm of copper (Cu) was vapor-deposited onto the Cr layer. The thickness was enough for the strain gauge but not for the part of the gauge that had to be soldered with wires that would be connected to the electronic circuit. To add the thickness of the metal at these parts, a stencil mask made from polyimide film was set on the strain gauge before the second deposition process of Cu. The thickness of the solder pads was set to be 350 nm. After the deposition process was complete, the photoresist was removed from the film using acetone. Then, an X shape was cut in the center of the film so that all of the strain gauges were isolated from each other, as pictured in Figure 5a.

To assemble the device, stainless-steel pole that is 1 mm in diameter and 10 mm in height was first placed in the center of the acrylic base’s hole. A silicone elastomer paste (Dow Corning 3145 RTV MIL-A-46146 adhesive/sealant, Dow Corning Corp., Midland, MI, USA) was used as the glue between the base and the pole. This adhesive paste was elastic when cured so that when the load was applied to the sides of the pole, the pole would slant and then spring back to its initial position, i.e., 90° from the base, when the applied load was removed. The pole was set with the acrylic pole aligner, and the silicone elastomer paste was left for 24 h in a desiccator. Next, the strain gauges were set in the middle of the acrylic base using a double-sided tape. The tip of the pole was inserted from the back of the strain gauge film through the aforementioned X-shaped cut. All of the four strain gauges were made sure to lean towards the pole and curled upwards. Figure 5b,c shows the photograph of the fabricated device. Measurement using a multimeter showed the initial resistance of the strain gauges as 42.6 ± 12.9 Ω.

## 4. Experimental Methods and Procedures

Each strain gauge of the device was connected to a resistor of 100 Ω in serial. By measuring the voltage across the resistor *V*_100_ and the strain gauge *V*_sg_ using a high-speed analog measurement system (Keyence NR-500 series, Keyence Corp., Osaka, Japan), the resistance of strain gauge *R*_sg_ was calculated as follows:

(1) $$R_{\mathrm{sg}}=\frac{V_{\mathrm{sg}}}{V_{100}}R_{100}$$

**Figure 6:**
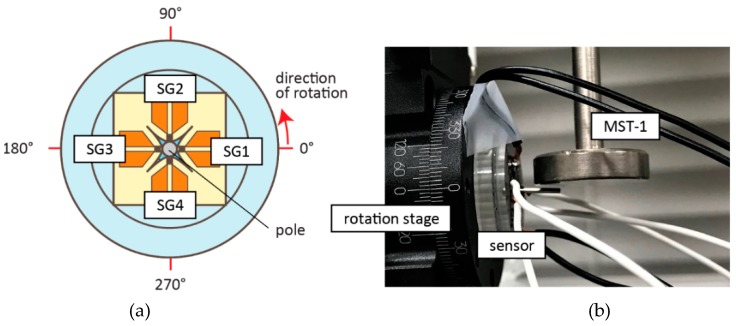
The experiment procedures. (**a**) Definition of the angle at which the load was applied. (**b**) The device was set on a rotation stage and the load applied to the sides of the device with micro strength evaluation testing machine (MST-I). The direction of rotation from 0° to 360° during the experiment, with load applied every 15°.

The sensitivity of the sensor was determined by the proportional coefficient between the ratio of the resistance change and the applied load or the displacement of the pole. We defined the angle of zero when the load was applied from the strain gauge SG1, as shown in Figure 6a. The experiment was conducted with the load being applied to the side of the center pole at every 15° using a micro strength tester (Micro Autograph MST-I, Shimadzu Corporation, Kyoto, Japan), as shown in Figure 6b. The output signal of the load, displacement, and voltage were recorded and analyzed.

An experiment was also conducted to test the loading repeatability and durability of the device. Considering the application to MIS, in which the sensor scans at low speed, the load was added to the side of the center pole of the device repetitively, at 0.05 Hz to 0.25 Hz, 10 times. The amplitudes of the resistance change were recorded for each frequency, and the consistency of the resistance change were observed and analyzed. 

## 5. Experimental Results and Discussions

### 5.1. Sensitivity Test

**Figure 7:**
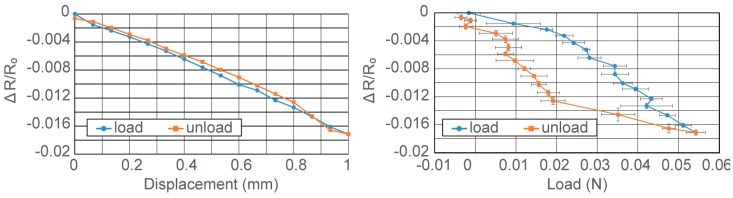
Results for the sensitivity tests. (**a**) Change of resistance as compared to displacement of center pole during load application, and (**b**) change of resistance as compared to the applied load.

Figure 7 shows the result of the sensitivity test for the fabricated device. It shows the average and the standard deviation as the error bar among four trials, each including a load and unload cycle. As shown in Figure 7a, resistance change ratios decreased with the increase of the displacement of the cylindrical load, 5 mm from the tip of the pole. Little hysteresis was observed in the experiments. The resolution of the sensor was 0.067 mm of displacement, while sensitivity of 0.016 mm^−1^ for the resistance change per 1 mm displacement was obtained. Figure 7b shows the relationship between the applied load and the resistance change ratio. Compared to Figure 7a, increased hysteresis was observed in Figure 7b. We consider this due to the viscoelastic properties of the elastomer paste that was used as the glue at the bottom of the center pole. A resolution of 0.002 N and sensitivity of 0.313 N^−1^ for resistance change per 1 N load applied to the center pole was obtained. Figure 7b indicated some abnormal resistance variations at 0.03 to 0.05 N during loading, and less than 0.01 N during unloading, which was likely due to a slip between the pole and the tip of the triangular strain gauge film.

As mentioned in Section 2, the proposed sensor device had a superiority in design and fabrication of the device, where the sensor elements were in three-dimensional form and elastically balanced. The information from all sensor devices was taken into account during application, thus the hysteresis was counterweighed.

### 5.2. Direction of Load

**Figure 8:**
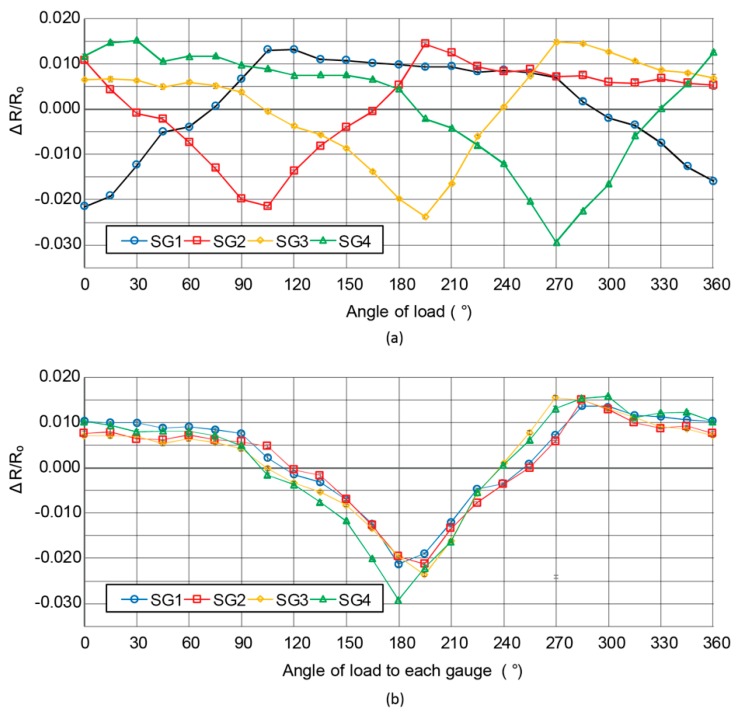
Results of the test for direction of load recognition. (**a**) The direction of rotation from 0° to 360° and the ratio of resistance change for each degrees of the same load applied. The results were replotted for 0° defined as the opposite of each strain gauge as shown in (**b**) the replotted graph.

Figure 8 shows the relationship between the ratio of the resistance change and the direction of the load applied to the center pole. From the plotted graph in Figure 8a, the trend for each strain gauge was concluded as a sine curve, with a nearly constant resistance change ratio for several angles. For better understanding about the ratio of resistance change and the direction of load from the position of strain gauge, the results in Figure 8a were replotted in Figure 8b, with 0° defined as the opposite position of each strain gauge. We saw good consistency among the four strain gauges, which verified the elastic balancing of the center pole worked as we expected. Observing from the position of the strain gauges and the direction of load, the resistance change ratio was at the lowest point between 180° and 195° and the highest point at 270° to 300°. The values of resistance change ratios were observed to be almost constant, but began slightly decreasing from 315° to 330°, and ended at 75° to 105° from each strain gauge before they decreased gradually with the increase of the angles. 

The resistance change ratio was reasonably the lowest when the load was applied from the side of the strain gauge, i.e., in the direction to reduce the bending moment to the gauge. The highest ratio was observed from 270° to 300° because the circuits including the strain gauges possessed asymmetry in wiring. On the contrary, given that the strain gauges were already compressed when the center pole was inserted, the sensitivity of each strain gauge in the direction of compression was not high. The consistency of the pattern of the experimental results verified effectiveness of the proposed assembly process, i.e., elastic balancing of the center pole. 

### 5.3. Loading Repeatibility Test

**Figure 9:**
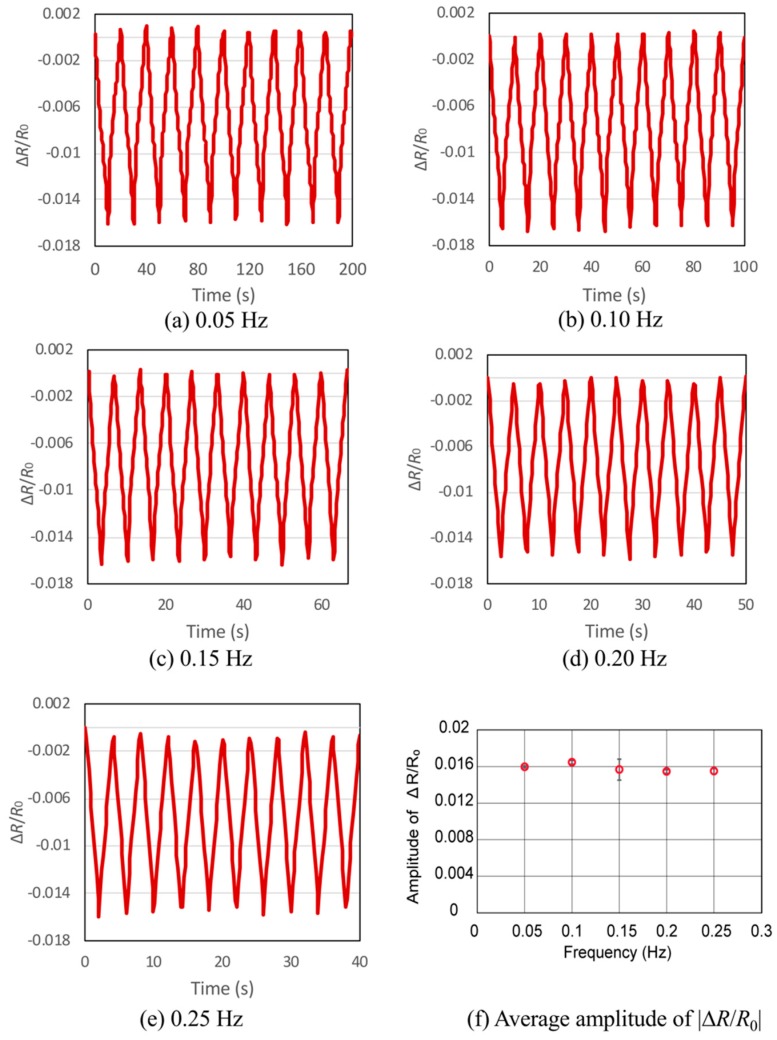
Results for loading repeatability test for cyclic loading frequency of (**a**) 0.05 Hz, (**b**) 0.10 Hz, (**c**) 0.15 Hz, (**d**) 0.20 Hz, (**e**) 0.25 Hz, and (**f**) the absolute values of average amplitude of resistance change ratios for all frequencies. The amplitudes of resistance change ratio were consistent for the loading frequency of 0.05 Hz to 0.25 Hz.

When the load was added to one side of the center pole repetitively, the results plotted in Figure 9 clearly show that the changing of loading frequency did not affect the amplitudes of the resistance change ratio. It was observed in Figure 9a–e that there were no significant delays in the sensor’s response during the cyclic loading test. In the application of MIS, scanning speed of the sensor is not considered to be high, thus this sensor would be sufficient for such applications.

## 6. Conclusions

A tactile sensor device mimicking the anatomy of a tooth was successfully demonstrated. The device was fabricated with four strain gauges surrounding a stainless-steel center pole. The assembly process of inserting the pole into the center of the flexible substrate with the gauges was proposed and later verified by the experiments, where all four strain gauges showed good consistency. The bottom of the center pole was fixed with a silicon adhesive paste, of which viscoelasticity caused a hysteresis in load/unload tests. The device showed a consistent response for all strain gauges with a sine-curve pattern and some constant change through all 360° of load direction. The tooth-inspired tactile sensor proposed herein did detect the amplitude and direction of the applied load. Given the tactile sensor’s simple structure, it could be readily applicable to MIS with further miniaturization.
